# Supplementary material for: Interplay between MIG-6/papilin and TGF-β signaling promotes extracellular matrix remodeling and modulates the maintenance of neuronal architecture
Source: bioRxiv. 2025 May 8:2025.05.08.652809. Preprint. [Version 1] doi: 10.1101/2025.05.08.652809 (PMC12247666; doi:10.1101/2025.05.08.652809)
Supplement: 1 [file NIHPP2025.05.08.652809V1-supplement-1.pdf]

bioRxiv preprint doi: https://doi.org/10.1101/2025.05.08.652809; this version posted May 8, 2025. The copyright holder for this preprint (which was not certified by peer review) is the author/funder, who has granted bioRxiv a license to display the preprint in perpetuity. It is made available under aCC-BY 4.0 International license.

Table S1. List of strains used.

Strain	Genotype	Transgene	Reference
N2			[1]
VH648	*hdIs26 III*	*Podr-2::cfp; Psra-6::DsRed2*	[2]
VQ1742	*qvIs9 II*	Integration of *dzEx1622 [Pmig-6::mNeonGreen::mig-6S]*. *dzEx1622* described in [3]	This study
NK245	*qyIs7 X*	*lam-1::gfp*	[4]
NK364	*unc-119(ed3) III; qyIs46 X*	*Pemb-9::emb-9::mCherry; unc-119(+)*	[5]
NK2565	*pxn-2(qy76) X*	*mNeonGreen::pxn-2 N-term*	[6]
NK2579	*fbl-1(qy62) IV*	*mNG+loxP::fbl-1*	[6]
BW1940	*ctIs40 X*	*dbl-1(+) + Psur-5::GFP*	[7]
TLG182	*texIs100 IV*	*Pdbl-1::dbl-1:gfp ; Pttx-3::rfp*	[8]
NU3	*dbl-1(nk3) V*		[9]
LT186	*sma-6(wk7) II*		[10]
RJP1553	*sma-6(wk7) II; zdIs13 IV; yxEx615*	*yxEx615 [Psma-6::sma-6; Punc-122::gfp]*	[11, 12]
CS152	*sma-3(wk30) III; qcIs6 [GFP::SMA-3 + rol-6(d)]*		[13]
**Neuronal phenotype characterization**			
VQ1061	*sax-7(qv30) IV; hdIs26 III*		[14]
VQ1076	*mig-6(qv33) V; hdIs26 III*		[14]
VQ1077	*sax-7(qv30) IV; mig-6(qv33) V; hdIs26 III*		[14]
VQ1828	*sma-6(wk7) II; hdIs26 III*		This study
VQ1852	*sma-6(wk7) II; mig-6(qv33) V; hdIs26 III*		This study
VQ1866	*sma-6(wk7) II; sax-7(qv30) IV; hdIs26 III*		This study
VQ1905	*sma-6(wk7) II; sax-7(qv30) IV; mig-6(qv33) V; hdIs26 III*		This study
VQ1932	*sax-7(qv30) IV; mig-6(qv33) V; hdIs26 III; ctIs40 X*		This study
VQ1933	*sax-7(qv30) IV; hdIs26 III; ctIs40 X*		This study
VQ1934	*mig-6(qv33) V; hdIs26 III; ctIs40 X*		This study
VQ1935	*hdIs26 III; ctIs40 X*		This study
**Tissue-specific rescue assays**			
VQ2024	*sax-7(qv30) IV; mig-6(qv33) V; hdIs26 III; qvEx632*	*Pelt-3::sma-6::gfp*	This study
VQ2096	*sax-7(qv30) IV; mig-6(qv33) V; hdIs26 III; qvEx667*	*Pelt-3::sma-6::gfp*	This study
VQ2097	*sax-7(qv30) IV; mig-6(qv33) V; hdIs26 III; qvEx668*	*Pelt-3::sma-6::gfp*	This study
VQ2044	*sax-7(qv30) IV; mig-6(qv33) V; hdIs26 III; qvEx641*	*Prab-3::sma-6*	This study
VQ2045	*sax-7(qv30) IV; mig-6(qv33) V; hdIs26 III; qvEx642*	*Prab-3::sma-6*	This study
VQ2046	*sax-7(qv30) IV; mig-6(qv33) V; hdIs26 III; qvEx643*	*Prab-3::sma-6*	This study
VQ2075	*sax-7(qv30) IV; mig-6(qv33) V; hdIs26 III; qvEx653*	*Pmyo-3::sma-6*	This study
VQ2076	*sax-7(qv30) IV; mig-6(qv33) V; hdIs26 III; qvEx654*	*Pmyo-3::sma-6*	This study
VQ2077	*sax-7(qv30) IV; mig-6(qv33) V; hdIs26 III; qvEx655*	*Pmyo-3::sma-6*	This study

bioRxiv preprint doi: https://doi.org/10.1101/2025.05.08.652809; this version posted May 8, 2025. The copyright holder for this preprint (which was not certified by peer review) is the author/funder, who has granted bioRxiv a license to display the preprint in perpetuity. It is made available under aCC-BY 4.0 International license.

**Control strains for tissue-specific rescue assays**			
VQ2071	*hdIs26 III; qvEx650*	*Pelt-3::sma-6::gfp*	This study
VQ2073	*hdIs26 III; qvEx652*	*Pelt-3::sma-6::gfp*	This study
**EMB-9/Collagen IV expression pattern**			
VQ1776	*mig-6(qv33) V; emb-9(qy24) III*		[14]
VQ1843	*sma-6(wk7) II; emb-9(qy24) III*		This study
VQ1176	*mig-6(qv33) V; qyIs46 X*		[14]
VQ1795	*sma-6(wk7) II; qyIs46 X*		This study
VQ1839	*sma-6(wk7) II; mig-6(qv33) V; qyIs46 X*		This study
VQ1997	*qyIs46 X; yxEx615*	*Psma-6::sma-6*	This study
**PXN-2/Peroxidasin expression pattern**			
VQ1547	*mig-6(qv33) V; pxn-2(qy76) X*		[14]
VQ1938	*sma-6(wk7) II; pxn-2(qy76) X*		This study
VQ1946	*sma-6(wk7) II; mig-6(qv33) V; pxn-2(qy76) X*		This study
VQ2034	*pxn-2(qy76) X; yxEx615*	*Psma-6::sma-6*	This study
VQ2035	*mig-6(qv33) V; pxn-2(qy76) X; yxEx615*	*Psma-6::sma-6*	This study
**FBL-1/Fibulin expression pattern**			
VQ1837	*mig-6(qv33) V; fbl-1(qy62) IV*		This study
VQ1904	*sma-6(wk7) II; fbl-1(qy62) IV*		This study
VQ1906	*sma-6(wk7) II; mig-6(qv33) V; fbl-1(qy62) IV*		This study
VQ2113	*fbl-1(qy62) IV; yxEx615*	*Psma-6::sma-6*	This study
**LAM-1/Laminin expression pattern**			
VQ1175	*mig-6(qv33) V; qyIs7 X*		This study
VQ1876	*sma-6(wk7) II; qyIs7 X*		This study
VQ1880	*sma-6(wk7) II; mig-6(qv33) V; qyIs7 X*		This study
VQ2152	*qyIs7 X; yxEx615*	*Psma-6::sma-6*	This study
**MIG-6S/Papilin expression pattern**			
VQ1998	*dbl-1(nk3) V; qvIs9 II*		This study
VQ2013	*qvIs9 II; yxEx615*	*Psma-6::sma-6*	This study
**DBL-1 expression pattern**			
VQ1971	*mig-6(qv33) V; texIs100 IV*		This study
**GFP::SMA-3**			
VQ1996	*qcIs6*		This study
VQ2017	*mig-6(qv33) V; qcIs6*		This study

bioRxiv preprint doi: https://doi.org/10.1101/2025.05.08.652809; this version posted May 8, 2025. The copyright holder for this preprint (which was not certified by peer review) is the author/funder, who has granted bioRxiv a license to display the preprint in perpetuity. It is made available under aCC-BY 4.0 International license.

1. Brenner S. The genetics of *Caenorhabditis elegans*. Genetics. 1974;77(1):71-94.
2. Hutter H. Extracellular cues and pioneers act together to guide axons in the ventral cord of *C. elegans*. 2003.
3. Ramirez-Suarez NJ, Belalcazar HM, Salazar CJ, Beyaz B, Raja B, Nguyen KC, et al. Axon-dependent patterning and maintenance of somatosensory dendritic arbors. Developmental cell. 2019;48(2):229-44. e4.
4. Hagedorn EJ, Yashiro H, Ziel JW, Ihara S, Wang Z, Sherwood DR. Integrin acts upstream of netrin signaling to regulate formation of the anchor cell's invasive membrane in *C. elegans*. Dev Cell. 2009;17(2):187-98. doi: 10.1016/j.devcel.2009.06.006. PubMed PMID: 19686680; PubMed Central PMCID: PMCPMC2729287.
5. Ihara S, Hagedorn EJ, Morrissey MA, Chi Q, Motegi F, Kramer JM, Sherwood DR. Basement membrane sliding and targeted adhesion remodels tissue boundaries during uterine–vulval attachment in *Caenorhabditis elegans*. Nature cell biology. 2011;13(6):641-51.
6. Keeley DP, Hastie E, Jayadev R, Kelley LC, Chi Q, Payne SG, et al. Comprehensive endogenous tagging of basement membrane components reveals dynamic movement within the matrix scaffolding. Developmental cell. 2020;54(1):60-74. e7.
7. Suzuki Y, Yandell MD, Roy PJ, Krishna S, Savage-Dunn C, Ross RM, et al. A BMP homolog acts as a dose-dependent regulator of body size and male tail patterning in *Caenorhabditis elegans*. Development. 1999;126(2):241-50.
8. Schultz RD, Bennett EE, Ellis EA, Gumienny TL. Regulation of extracellular matrix organization by BMP signaling in *Caenorhabditis elegans*. PloS one. 2014;9(7):e101929.
9. Morita K, Chow KL, Ueno N. Regulation of body length and male tail ray pattern formation of *Caenorhabditis elegans* by a member of TGF-β family. Development. 1999;126(6):1337-47.
10. Krishna S, Maduzia LL, Padgett RW. Specificity of TGFβ signaling is conferred by distinct type I receptors and their associated SMAD proteins in *Caenorhabditis elegans*. Development. 1999;126(2):251-60.
11. Zhang X, Zhang Y. DBL-1, a TGF-β, is essential for *Caenorhabditis elegans* aversive olfactory learning. Proceedings of the National Academy of Sciences. 2012;109(42):17081-6.
12. Baltaci O, Pedersen ME, Sherry T, Handley A, Snieckute G, Cao W, et al. Atypical TGF-β signaling controls neuronal guidance in *Caenorhabditis elegans*. Iscience. 2022;25(2).
13. Wang J, Tokarz R, Savage-Dunn C. The expression of TGFbeta signal transducers in the hypodermis regulates body size in *C. elegans*. Development. 2002;129(21):4989-98. doi: 10.1242/dev.129.21.4989. PubMed PMID: 12397107.
14. Nadour M, Valette R. L. RI, Biard M, Frebault N, Rivollet L, St-Louis P, et al. Remodeling of extracellular matrix collagen IV by MIG-6/papilin regulates neuronal architecture. bioRxiv. 2025. Epub 20250210. doi: 10.1101/2025.02.10.637428. PubMed PMID: 39990436; PubMed Central PMCID: PMCPMC11844411.

bioRxiv preprint doi: https://doi.org/10.1101/2025.05.08.652809; this version posted May 8, 2025. The copyright holder for this preprint (which was not certified by peer review) is the author/funder, who has granted bioRxiv a license to display the preprint in perpetuity. It is made available under aCC-BY 4.0 International license.

Table S2. List of primers used.

Gene	Primer	Sequence	PCR product (bp)	Note
sax-7(qv30)				
Mutant specific	oCB747	TCTCTCAAAATTCTTCGCAAGC	336	
	oCB1025	CGGGAAGAAATGAAACAGGA		
Wild-type specific	oCB212	GAAATACACACAAATACGAGTGC	592	
	oCB723	TAGTTGATTAAAATGTTTCAAGATTG		
mig-6(qv33)				
	oCB2241	CTCCCAAGGAAGAGCCTATCC	586	BamHI digestion:
	oCB2242	CGAGCAGTTAGAGCATCCG		Mutant, band 586 bp<br>WT, two bands at 203 and 383 bp
